# The effect of dexmedetomidine on rocuronium-induced neuromuscular blockade and its reversal by sugammadex

**DOI:** 10.1186/s40635-025-00850-9

**Published:** 2026-01-07

**Authors:** Marianna Fedor, Nikolett Sallai, Béla Fülesdi, Ákos I. Fábián

**Affiliations:** Department of Anesthesiology and Intensive Care, Faculty of Medicine, University of Debrecen, Debrecen, Hungary

**Keywords:** Dexmedetomidine, Phrenic nerve–diaphragm preparation, Neuromuscular block, Rocuronium, Sugammadex

## Abstract

**Background:**

Dexmedetomidine (DEX) is increasingly used in the intensive care unit for sedation and also serves as an adjuvant in general anesthesia and in procedural sedations. We tested whether dexmedetomidine at different concentrations influences the activity of the neuromuscular junction at the diaphragm and whether DEX has an impact on the action of rocuronium at the diaphragm as well as the reversal of the neuromuscular block by sugammadex.

**Methods:**

20 male Wistar rat phrenic nerve–hemidiaphragm system was used for our experiments. The concentration–response characteristics of DEX and rocuronium were quantified as the depression of the force amplitude of single twitches (ST) in response to electrical stimulation of the phrenic nerve. Rocuronium concentration–response curves were obtained with 0, 1, and 2.67 μg/ml DEX concentration. After a single dose of rocuronium, sugammadex doses were given until additional doses of sugammadex were not accompanied by a further increase in ST force amplitude. The concentration–response curve of sugammadex was also measured in the presence of 1 μg/ml DEX concentration.

**Results:**

DEX at different doses negligibly reduces the force of the contractions and the contractility of the diaphragm. The EC50 of rocuronium [7.74 µM (6.99–8.57)] did not change significantly [7.18 µM (6.58–7.84); *p* = 0.27] with the addition of DEX 1 µg/ml. At 2.67 µg/ml DEX concentration, the ED50 of rocuronium was significantly reduced [6.37 µM (5.69–7.13); *p* = 0.015]. With 1 µg/ml DEX concentration, the EC50 of the sugammadex [2.04 µM (1.94–2.14)] needed for the reversal of rocuronium-induced neuromuscular blockade was significantly increased [2.45 µM (2.39–2.51); *p* < 0.01].

**Conclusions:**

DEX at clinically administered doses does not significantly influence the function of the neuromuscular junction at the diaphragm. Under routine dosing conditions, the action of the neuromuscular blocking agents and their reversal by sugammadex are also not modified by DEX.

**Supplementary Information:**

The online version contains supplementary material available at 10.1186/s40635-025-00850-9.

## Introduction

The alpha-2 receptor agonist dexmedetomidine (DEX) is increasingly used in the intensive care unit (ICU) for sedation to reduce patient–ventilator asynchrony and minimize delirium and agitation [1]. It inhibits the release of norepinephrine from the nucleus coeruleus and in the posterior horn of the spinal cord, which facilitates a calm, anxiolytic, and mild analgesic sedation. Recent international guidelines and meta-analyses proposed a conditional recommendation favoring the use of dexmedetomidine over benzodiazepines [2]. Dexmedetomidine, when used for light sedation in the intensive care unit, ensures shorter invasive ventilation and ICU stay over propofol sedation [3]. In the past decade, dexmedetomidine also served as an adjuvant in general anesthesia and in procedural sedations [4–7].

Besides the known and frequently observed side effects of dexmedetomidine (hypotension and bradycardia), animal reports suggest that it may cause muscle flaccidity and decrease electromyographic activity [8]. It has been shown that alpha-adrenoceptors are also located at the neuromuscular junction and may play a role in neuromuscular transmission [9, 10], indicating that dexmedetomidine may influence muscle function through a peripheral, rather than a central effect. Whether DEX influences the function of the neuromuscular junction is important to know because this may influence the function of the diaphragm during long-term sedation for mechanical ventilation in the ICU, and it may also influence the action and reversal of neuromuscular blocking agents in the anesthesiology setting. Data on the effect of DEX on the neuromuscular junction are scarce. An experimental study using vecuronium could not prove an effect of dexmedetomidine on the function of the neuromuscular junction [11]. In a clinical study in humans, the plasma rocuronium concentration increased and the T1 time in the train-of-four (TOF) sequence decreased during administration of DEX, but not in a clinically significant manner [12].

In the present animal study, we aimed to assess whether dexmedetomidine at different concentrations influences the activity of the neuromuscular junction at the diaphragm. In a further part, we also tested whether DEX has an impact on the action of rocuronium at the diaphragm and the reversal of the neuromuscular block by sugammadex.

## Methods

### Animals

Ethics approval for this study (1/2013/DE MÁB) was provided by the University of Debrecen Committee of Animal Research, Debrecen, Hungary (Chairperson Prof I. Furka) on 15 April 2013. A total of 20 male Wistar rats, ranging in weight from 250 to 563 g, were used. Institutional guidelines for animal care and usage for research principles were strictly followed. Animals were chosen randomly on the morning of the experiment and killed before harvesting tissue specimens.

### Materials

Rocuronium (Esmeron; MSD Pharma Hungary, Budapest, Hungary), sugammadex (Bridion; MSD Pharma Hungary, Budapest, Hungary), and dexmedetomidine (Dexdor, Orion Corporation, Espoo, Finland) were purchased from commercial vendors and diluted in Krebs buffer as needed to achieve a dosing volume of 10–100 µl.

### Experimental procedures

The rat phrenic nerve–hemidiaphragm system was used for our experiments. Rats were killed with an intraperitoneal overdose of sodium thiopental (60 mg/kg) and exsanguinated through the incision of the dorsal vena cava. Hemidiaphragm preparation was performed by using a modified version of the technique originally described by Bülbring. Briefly, bilateral thoracotomy and removal of the sternum were performed, after which both phrenic nerves were dissected from cranial to rostral direction to the diaphragmatic insertion. Then both hemidiaphragms were excised with the corresponding phrenic nerve intact. The hemidiaphragms were then secured in a phrenic nerve–diaphragm tissue holder (ISO-07-TSZ2D, Experimetria Ltd., Budapest, Hungary) in 75 mL of Krebs buffer (110 mM NaCl, 5 mM KCl, 1.25 mM CaCl_2_, 1 mM MgSO_4_, 1 mM KH_2_PO_4_, 5 mM glucose, 20 mM NaHCO_3_) aerated by bubbling 95% O_2_ + 5% CO_2_ (Vol%) through the solution. The solution was maintained at a temperature of 37 °C (AMP-09 Temperature controller, Experimetria Ltd., Budapest, Hungary).

The hemidiaphragms were attached to an isometric force–displacement transducer (FSG-01/200 Force Transducer, Experimetria Ltd., Budapest, Hungary) at the diaphragmatic centrum tendineum using a commercially available 5/0 diameter surgical suture. Measurements were amplified by an AMP-01-SG Classic bridge amplifier and recorded with a 16-channel professional software package (S.P.E.L. Advanced Isosys software, Experimetria Ltd., Budapest, Hungary). The phrenic nerve was stimulated either with a single twitch every 5 s (rectangular pulses of 0.3 ms pulse width and supramaximal voltage) or a 2-Hz train-of-four (TOF) stimulus every 15 s (rectangular pulses of 0.2 ms duration with a supramaximal voltage) using a square wave stimulator (ST-03-O4, Experimetria Ltd., Budapest, Hungary).

After submersion in the buffer solution, the tissue preparations were given a 10-min acclimatization period without stimulation at an applied resting tension of 20–30 mN. Then, stimulation was initiated, followed by an additional 1–1.5 h without treatment (with buffer changes as needed) until a stable baseline tension was reached. Dex, rocuronium, and sugammadex dosing were only started after this stabilization period. After measuring a given concentration–response curve, the buffer solution was exchanged five times in a 30-min timespan to ensure the complete washout of any agents before measuring a new concentration–response curve.

Multiple measurements were performed on any given specimen; however, one specimen contributed only one data set to any given concentration–response curve. To mitigate the effects of degradation of the tissue specimen over time, the order of concentration–response curve experiments was permuted between specimens. A specimen was discarded if a stable baseline tension was no longer attainable. Each concentration–response relationship shown in the figures is based on concentration–response curves from 5 specimens.

The concentration–response characteristics of DEX and rocuronium were quantified as the depression of the force amplitude of single twitches (ST) in response to electrical stimulation of the phrenic nerve (from here on referred to as ST force amplitude). Drug doses were given in 15-min intervals. The ST force amplitude at a given drug concentration consisted of the mean value of five consecutive contractions corrected with the baseline tension measured between contractions, measured once the contraction amplitude had stabilized and did not visibly change over time. The ST force amplitude was normalized to the maximum contraction amplitude of the untreated sample to construct cumulative concentration–response curves. Each specimen contributed 5–8 measurement points to the curve.

Each hemidiaphragm preparation was typically used for 2–3 concentration–response curves over a total experimental duration of up to 6 h. Baseline tension between contractions served as a continuous indicator of tissue viability and was numerically recorded. Preparations were discarded when a stable baseline tension could no longer be maintained, based on clear visual evidence of sustained baseline drift and/or a progressive decline in twitch force that did not recover after washout. These changes were verified by post hoc inspection of the numerically recorded data. To assess intrinsic time-dependent drift, a separate time-control experiment was performed in which a preparation was monitored for 2 h without solution change or drug exposure, and no significant change in contraction force was observed. All concentration–response curves were normalized to the initial stable supramaximal twitch amplitude recorded at the beginning of each individual curve. This curve-specific normalization minimized the influence of slow time-dependent drift across the experimental day.

Treatment sequence was assigned in an informal randomized manner prior to experimentation for each preparation. Because not all preparations remained viable for the full experimental duration, not all specimens contributed data to all three treatment conditions. Full blinding was not feasible, as the same investigator performed tissue preparation, stimulation, solution preparation, and data acquisition. Treatment times and drug additions were automatically logged by the electronic recording system. Objective numerical criteria (baseline tension stability, twitch amplitude preservation, and normalization to the start–of–curve baseline) governed data inclusion.

### Dosing interval and quasi-steady state

Drug concentrations were applied at 15-min intervals. This timing was based on pilot experiments performed in the same preparation. Following single large doses, maximal effects were typically reached within approximately 20–25 min. During incremental concentration–response experiments, twitch amplitude exhibited an initial faster change followed by gradual stabilization, reaching a visually stable plateau within approximately 15 min. Continuous twitch recording was performed throughout the experiments. If a visually stable plateau was not achieved at 15 min, the subsequent dose was delayed until stability was observed. These conditions support the attainment of a quasi-steady pharmacodynamic state at each concentration step.

### Washout procedure

Washout was performed by repeated complete exchange of the bathing solution. Typically, 3–4 full solution changes were carried out over approximately 20 min to ensure effective drug removal from the bath and tissue. A transient increase in twitch amplitude was consistently observed immediately after fresh solution exchange, followed by stabilization. After washout, contraction amplitude reliably returned toward baseline, indicating reversibility of drug effects.

To assess DEX interaction with rocuronium, rocuronium concentration–response curves were obtained as described above with 0, 1, and 2.67 μg/mL DEX concentration in the buffer solution. In isolated organ bath preparations, nominal drug concentrations cannot be directly equated to in vivo plasma levels due to the absence of circulation, tissue distribution gradients, plasma protein binding, and metabolic clearance. Consequently, higher bath concentrations are commonly required to ensure effective tissue-level receptor exposure. The applied dexmedetomidine concentrations therefore represent supraclinical in vitro exposure levels selected to ensure adequate tissue penetration and to explore the maximal potential peripheral effects of α2-adrenoceptor activation at the neuromuscular junction.

To measure the concentration–response curve of sugammadex, a single dose of rocuronium was given to achieve a 90–95% depression of ST force amplitude. Then sugammadex doses were given in 15-min intervals until additional doses of sugammadex were not accompanied by a further increase in ST force amplitude. A successful reversal (antagonism) was verified by measuring a TOF ratio [defined as the ratio between the fourth twitch response, T4, and the first twitch response, T1 (T4/T1) of the four stimuli] > 90%. The concentration–response curve of sugammadex was also measured in the presence of 1 μg/ml DEX concentration in the buffer solution to elucidate the potential effect of DEX on the reversal of neuromuscular block with sugammadex.

### Statistics

GraphPad Prism 6 for Windows (GraphPad Software, Inc., La Jolla, CA, USA) was used to fit concentration–response curves. Curve fitting was done by nonlinear regression with either the “log(agonist) vs. normalized response–variablef slope” or the “log(inhibitor) vs. normalized response–variable slope” method. The fitting equation was: y = 100/(1 + 10^((logEC50-X)*HillSlope)), where X is the log_10_ value of concentration, and y is the normalized and baseline corrected contraction amplitude.

Because multiple concentration–response points originated from each preparation, datapoints within a preparation are not statistically independent. Data were therefore pooled within each treatment condition and analyzed at the level of the fitted curves using the extra sum-of-squares F-test to compare shared versus separate curve models, with logEC50 specified as the parameter accounting for the extra sum of squares. This approach evaluates curve-level differences and does not explicitly model inter-preparation variability.

Group size (*n* = 5 hemidiaphragm preparations per concentration–response relationship) was chosen a priori based on preliminary data from our laboratory and on previous studies using similar rat phrenic nerve–hemidiaphragm preparations, as a compromise between statistical sensitivity and limitation of animal use in accordance with the 3R principles. These preliminary data indicated that this sample size would allow detection of moderate-to-large shifts in EC50 between treatments, while smaller differences cannot be definitively excluded. Statistical comparison of concentration–response curves was done with GraphPad Prism 6 for Windows using the extra sum-of-squares *F*-test, with logEC50 specified as the model parameter accounting for the extra sum of squares. Results are presented as mean and 95% confidence interval (CI) unless otherwise specified.

Because multiple concentration–response points were obtained from each preparation, these points are not statistically independent. Concentration–response data were therefore pooled within each treatment group and analyzed at the level of the fitted curves using the extra sum-of-squares *F*-test. This approach compares the goodness-of-fit of shared versus separate curve models but does not explicitly model inter-preparation variability.

## Results

**Fig. 1:**
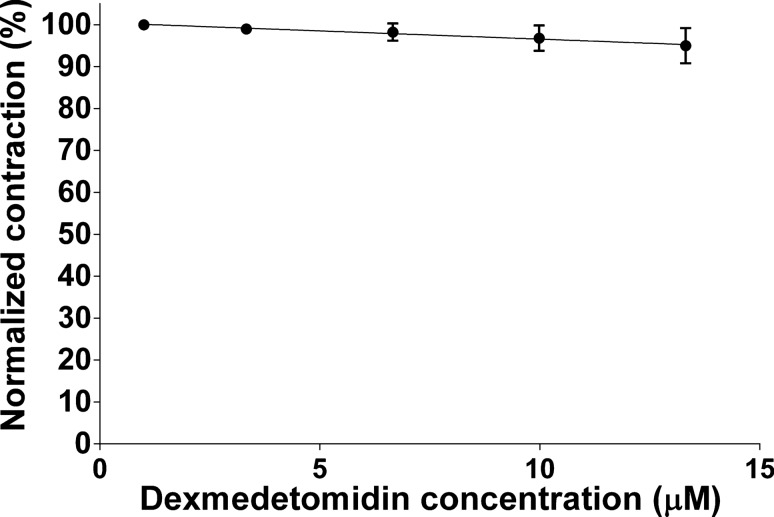
Contraction force after phrenic nerve stimulation at different concentrations of dexmedetomidine

The first step of our measurement was recording the changes in the force of the diaphragmatic contraction at increasing dexmedetomidine concentrations. We observed that dexmedetomidine negligibly reduces the force of the contractions and the contractility of the diaphragm (Fig. 1).

**Fig. 2:**
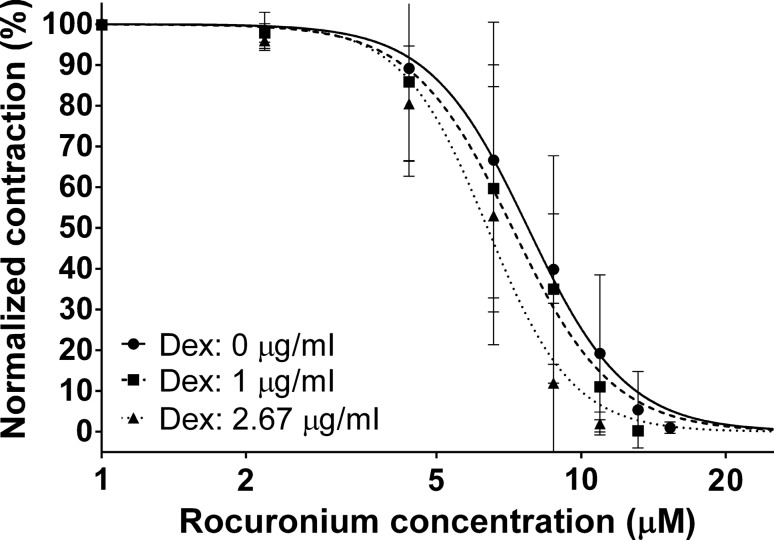
The impact of two different doses of dexmedetomidine on the neuromuscular blocking effect of rocuronium

At the concentrations we used, dexmedetomidine did not significantly enhance the neuromuscular depression caused by rocuronium. The EC50 of rocuronium [7.74 μM (6.99–8.57)] did not change significantly [7.18 μM (6.58–7.84); *p* = 0.27] with the addition of dexmedetomidine 1 μg/ml. We noticed that in the case of 2.67 μg/ml dexmedetomidine concentration, the ED50 of rocuronium was significantly reduced [6.37 μM (5.69–7.13); *p* = 0.015] (Fig. 2).

**Fig. 3:**
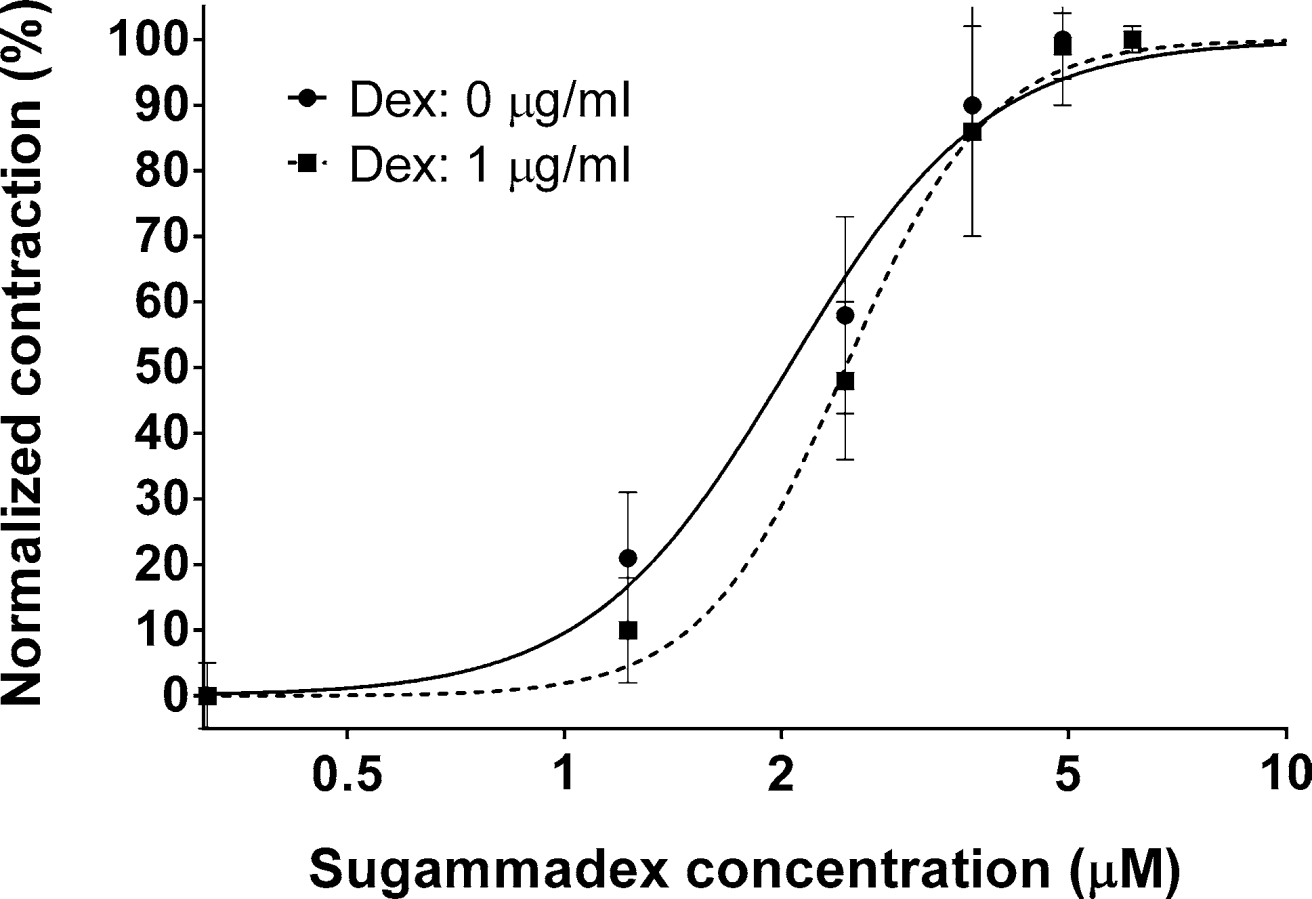
The effect of 1 μg/ml dexmedetomidine on the sugammadex concentration needed to completely reverse rocuronium-induced neuromuscular block

With 1 µg/ml dexmedetomidine concentration, the EC50 of the sugammadex [2.04 μM (1.94–2.14)] needed for the reversal of rocuronium-induced neuromuscular blockade was significantly increased [2.45 μM (2.39–2.51); *p* < 0.01] (Fig. 3).

## Discussion

In the present study, we found that even at a thousand times the clinically required dose [13], dexmedetomidine does not have an impact on neuromuscular transmission at the diaphragm. A further observation of the present study is that only supranormal concentrations of the drug result in prolongation of the neuromuscular blocking effect of rocuronium. Co-administration of dexmedetomidine necessitated the administration of higher doses of the reversal agent sugammadex.

Adrenoceptors play an important role in the regulation of the acetylcholine release at the neuromuscular junction. It has been shown that the facilitatory effect of adrenoceptors is manifested in the acetylcholine (ACh) quantal content, quantal size, and the sensitivity of the postsynaptic membrane to ACh [14]. This facilitating effect is linked to the alpha-1 adrenoceptors, and this is an important pathomechanism under stress situations when the speed and the force of the skeletal muscles’ contraction have to be increased. It is important to emphasize that the effect of sympathetic activation is different in fast-acting skeletal muscles and at the diaphragm, and this is due to the different fiber composition. The diaphragm contains slow, low-fatigue type I, fast oxygenation type II.a, and fast glycolytic type II.b fibers. In these muscles, the sympathetic activation occurs dominantly through alpha-2 adrenoceptors. In the skeletal muscles of the extremities, the regulation of the acetylcholine release is dominantly regulated through alpha-1 adrenoceptors. Stimulation of the alpha-2 receptors in this type of muscle does not result in any effect on postsynaptic membrane potential and end plate potential [15]. In contrast to this, in the diaphragm, activation of the alpha-2 receptors results in suppression of the quantal release of acetylcholine both at rest and after stimulation [16]. It has been shown that this effect is linked to the G-protein-gated inwardly rectifying potassium channel (GILK) activation, leading to inhibition of the voltage-gated Ca-channels and resulting in decreased acetylcholine release [17, 18]. Under normal dosing conditions, the administration of the alpha-2 adrenergic agonist dexmedetomidine may not affect the neuromuscular transmission at the diaphragm, as demonstrated in the present study. Although the quantal content of ACh was not measured in the present study, in previous experiments it has been demonstrated that dexmedetomidine is a potent stimulator of the ACh release and the exocytosis of the ACh vesicles at the presynaptic end plate [15]. It is conceivable that in clinical doses, stimulation of the alpha-2 receptors, although they decrease the quantal content of the released acetylcholine, is not sufficient to override the safety margin of the neuromuscular transmission [19]. This may explain why in the present study, lower doses of dexmedetomidine did not cause a significant decrease of the diaphragmatic contractions. After increasing the dose of DEX to a supraclinical level, a slight, but not significant, depression of the diaphragmatic contractions could be observed.

During the administration of a non-depolarizing neuromuscular blocking agent, there is an ongoing competition between the drug and acetylcholine at the postsynaptic endplate. If an alpha-2 agonist (such as dexmedetomidine) is administered during this competition, the release of acetylcholine decreases, which favors the action of the NMB agent at the postjunctional receptors. This may explain why neuromuscular function was not affected by dexmedetomidine during the administration of vecuronium in an animal experiment of Weinger and colleagues [11] and in human volunteers using rocuronium as an NMB agent [12]. In this human study, Talke and co-workers also observed an increased plasma concentration of rocuronium after the administration of dexmedetomidine, indicating that lower concentrations of rocuronium were sufficient to exert the same neuromuscular blocking effect. Similar to these observations, we also found that dexmedetomidine did not influence the neuromuscular blocking effect of rocuronium. At higher supraclinical doses of DEX, the ED50 dose of rocuronium significantly decreased, suggesting a further decrease in acetylcholine quantal content release.

A further finding of the present study is that dexmedetomidine in supraclinical doses significantly increases the ED50 dose of sugammadex during the reversal of neuromuscular block. The explanation for this observation may be again the decreased acetylcholine release caused by the alpha-2 agonist agent [15]. As the spontaneous release of acetylcholine at the presynaptic terminal is decreased, the competition between the acetylcholine and the muscle relaxant is shifted toward the neuromuscular blocking agent. Therefore, higher doses of sugammadex are needed to achieve the complete reversal of the neuromuscular block. However, because this occurs after administration of supraclinical doses of dexmedetomidine, this observation may not have implications in daily practice and is mainly of pathophysiological and pharmacokinetic value.

A limitation of the present study is that the nominal dexmedetomidine concentrations applied in the isolated hemidiaphragm preparation exceed plasma concentrations typically achieved during routine clinical administration. However, due to the absence of circulation, tissue distribution gradients, plasma protein binding, and metabolic clearance in the organ bath system, nominal bath concentrations cannot be directly translated to plasma exposure. Higher concentrations are therefore commonly required to achieve effective receptor engagement at the tissue level under static in vitro conditions. The supraclinical concentration was intentionally selected to define the upper mechanistic boundary of α2-adrenoceptor–mediated modulation at the neuromuscular junction. Importantly, even under these extreme exposure conditions, only modest effects on the pharmacodynamics of rocuronium and sugammadex were observed. Accordingly, these findings primarily provide mechanistic insight and should not be directly extrapolated to routine clinical dosing conditions. Given the group size and the observed variability of the concentration–response relationships, the study was primarily powered to detect moderate-to-large dexmedetomidine-induced changes in neuromuscular function, and the presence of smaller effects cannot be entirely excluded. A further limitation of the present study is that concentration–response curves were constructed from pooled data obtained from five independent preparations per condition. While this approach is appropriate for mechanistic curve-level comparisons, it does not explicitly quantify inter-preparation variability and therefore does not provide full population-level inference. Accordingly, the results should be interpreted primarily in terms of pooled pharmacodynamic behavior rather than preparation-specific effect size estimates. Although preparations were used for several hours, normalization to curve-specific baseline and continuous baseline tension monitoring limited the influence of slow time-dependent drift.

With respect to translational interpretation, even at exposure levels far exceeding those encountered clinically, dexmedetomidine did not meaningfully impair baseline neuromuscular transmission. Only supraclinical concentrations produced modest interactions with the pharmacodynamics of rocuronium and sugammadex. These effects therefore represent mechanistic upper-limit observations rather than direct clinical dose–effect relationships.

In conclusion, dexmedetomidine at concentrations exceeding those encountered under clinical conditions does not significantly impair neuromuscular transmission at the diaphragm. Only supraclinical exposure levels produced measurable modulation of the pharmacodynamic effects of rocuronium and its reversal by sugammadex. These findings suggest that dexmedetomidine is unlikely to exert clinically relevant peripheral neuromuscular effects under routine dosing conditions, while the observed interactions at extreme concentrations provide mechanistic insight into α2-adrenoceptor-mediated modulation of neuromuscular transmission.

## Supplementary Information

Supplementary Material 1.

Supplementary Material 2.

## Data Availability

The raw contraction force recordings, normalized concentration–response data, fitted curve parameters (logEC50, EC50, Hill slope), and statistical analysis files are available from the corresponding author upon reasonable request.
