# Supplementary material for: The effect of dexmedetomidine on rocuronium-induced neuromuscular blockade and its reversal by sugammadex
Source: Intensive Care Med Exp. 2026 Jan 7;14:2. doi: 10.1186/s40635-025-00850-9 (PMC12779884; doi:10.1186/s40635-025-00850-9)
Supplement: Supplementary file 1 — Supplementary Material 1. [file 40635_2025_850_MOESM1_ESM.docx]

| **Curve** | **LogEC50** | **EC50 (μM)** | **95% CI (μM)** | **Hill slope** | ***p* value**  **(compared to Rocuronium)** |
| --- | --- | --- | --- | --- | --- |
| Rocuronium | 0.89 | 7.74 | 6.99 – 8.57 | -4.23 | - |
| Rocuronium + Dex 1 μg/ml | 0.86 | 7.18 | 6.58 – 7.84 | -4.18 | 0.27 |
| Rocuronium + Dex 2.67 μg/ml | 0.80 | 6.37 | 5.69 – 7.13 | -4.90 | 0.015 |

| **Curve** | **LogEC50** | **EC50 (μM)** | **95% CI (μM)** | **Hill slope** | ***p* value**  **(compared to Sugammadex)** |
| --- | --- | --- | --- | --- | --- |
| Sugammadex | 0.31 | 2.04 | 1.94 – 2.14 | 3.14 | - |
| Sugammadex + Dex 1 μg/ml | 0.39 | 2.45 | 2.39 – 2.51 | 4.38 | 0.01< |
